# Recent Advances in Sensitized Photocathodes: From Molecular Dyes to Semiconducting Quantum Dots

**DOI:** 10.1002/advs.201700684

**Published:** 2018-01-08

**Authors:** Hao‐Lin Wu, Xu‐Bing Li, Chen‐Ho Tung, Li‐Zhu Wu

**Affiliations:** ^1^ Key Laboratory of Photochemical Conversion and Optoelectronic Materials Technical Institute of Physics and Chemistry The Chinese Academy of Sciences Beijing 100190 P. R. China; ^2^ School of Future Technology University of Chinese Academy of Sciences Beijing 100049 P. R. China

**Keywords:** molecular dyes, quantum dots, sensitized photocathodes, water splitting

## Abstract

The increasing demand for sustainable and environmentally benign energy has stimulated intense research to establish highly efficient photo‐electrochemical (PEC) cells for direct solar‐to‐fuel conversion via water splitting. Light absorption, as the initial step of the catalytic process, is regarded as the foundation of establishing highly efficient PEC systems. To make full use of visible light, sensitization on photoelectrodes using either molecular dyes or semiconducting quantum dots provides a promising method. In this field, however, there remain many fundamental issues to be solved, which need in‐depth study. Here, fundamental knowledge of PEC systems is introduced to enable readers a better understanding of this field. Then, the development history and current state in both molecular dye‐ and quantum dot‐sensitized photocathodes for PEC water splitting are discussed. A systematical comparison between the two systems has been made. Special emphasis is placed on the research of quantum dot‐sensitized photocathodes, which have shown superiority in both efficiency and durability towards PEC water splitting at the present stage. Finally, the opportunities and challenges in the future for sensitized PEC water‐splitting systems are proposed.

## 1 Introduction

Concerning the rapid consumption of fossil fuels and the emergence of consequent environmental problems, it is urgent for mankind to explore low‐cost, sustainable, and environmentally friendly energy sources.^1^, ^2^ Solar energy, one of the inexhaustible natural resources, has great potential to replace traditional energy forms of fossil fuels for its high‐power delivery. Theoretically, solar energy reaching the surface of Earth in 1 h is comparable to the whole energy consumption of the whole world in one year.^3^, ^4^ However, the intermittent property of solar irradiation makes solar energy difficult to utilize directly. To avoid this drawback, enormous efforts have been paid to convert solar energy to other kinds of usable energy forms, such as thermal energy, electric power, and chemical fuel, to acquire persistent and steady power supply.^5^, ^6^, ^7^, ^8^, ^9^, ^10^

Compared with converting solar energy into thermal energy, other energy forms, electricity and chemical fuel, are more desired and preferred. Currently, solar energy can be directly converted into electricity by using photovoltaics, which are mainly composed of semiconductor photoelectrodes, such as silicon.^11^ While the prominent shortcoming of this process is that the resulted electricity cannot be stored safely, massively, and steadily for a long time. Furthermore, the high cost also hampers its scalable utilization. Thus, converting solar energy into chemical fuel, easy to store and transport, appears to be an effective alternative of solar energy conversion and also has received extensive attention in recent years.^12^, ^13^ In this regard, Mother Nature has provided us with a good model to achieve this purpose.

In nature, photosynthetic organisms, such as green plants, cyanobacteria, and algae, can absorb sunlight by antenna system, a highly ordered organization of pigments, which results in the generation of spatially separated electron–hole pairs. Subsequently, the generated holes are captured by oxygen evolving complex within photosystem II (PS II) to realize water oxidation. As by‐products of the oxygen evolution reaction, the resulting electrons are transported to photosystem I (PS I) by an electron transporting chain and are thermodynamics sufficiently to be used for fuel generation with an assist of a second light‐harvesting process in PS I. Most photosynthetic organisms use these electrons to fix CO_2_ and produce carbohydrates or proteins as the main products, while some other micro‐organisms like cyanobacteria can synthesize molecular hydrogen with the aid of hydrogenase. By critically coupling subsystems of PS I and PS II together, as well as various redox cofactors, the photosynthetic process can run efficiently.^14^, ^15^

**Scheme 1:**
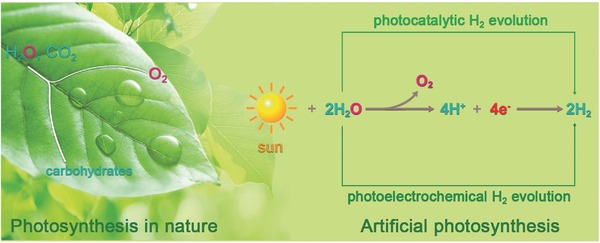
Illustration of main processes in converting solar energy into chemical fuels, especially hydrogen gas, of photosynthesis in nature and artificial photosynthesis.

Inspired by photosynthetic organisms, solar‐assisted water splitting system is established to mimic the process of natural photosynthesis, called as artificial photosynthesis, which has been considered as the “Holy Grail” for solar energy transformation (**Scheme** **1**).^16^, ^17^ Artificial photosynthesis can convert solar energy directly into chemical fuel in the form of chemical bonds within oxygen (O_2_), hydrogen (H_2_), and/or other carbohydrates, especially photoinduced water splitting as shown in Equation (1). With O_2_ as the single oxidation product, the only reduction product of this process is molecular H_2_, a carbon‐free and recyclable energy carrier with extremely high energy density, which is widely recognized as an ideal fuel in the future. Therefore, achieving high‐efficient solar light induced water splitting to produce H_2_ through artificial photosynthesis has drawn much attention.

(1) $$H_{2}O(l)\;\to \;H_{2}(g)\;+\;\frac{1}{2}O_{2}(g)\;\Delta G_{0}\;=\;+237\;\mathrm{kJ}\;\cdot {\mathrm{mol}}^{-1}$$

A significant breakthrough was achieved in 1972, when Fujishima and Honda realized the first light‐assisted water splitting by using an n‐type TiO_2_ semiconductor photoanode.^18^ Since then, various kinds of artificial photosynthetic systems with different materials have been established for sustainable and economical H_2_ generation with sunlight and water as the only inputs.^19^, ^20^, ^21^, ^22^, ^23^, ^24^ In general, artificial photosynthetic systems can be divided into two types: photocatalytic water splitting and photo‐electrochemical (PEC) water splitting.^25^, ^26^ Compared with photocatalytic counterparts, PEC systems demonstrate several unique superiorities, such as no sacrificial agents, ready separation of products, and the realization of overall water splitting,^27^, ^28^, ^29^, ^30^ which have made the development of highly efficient and robust PEC systems to be a very hot topic in recent years. Nonetheless, the p‐type PEC systems are mainly focused on molecular dye sensitized PEC systems, the quantum dot (QD) sensitized counterparts, as an emerging hot research area, have never been reviewed systematically. In this review, we have made, for the first time, a detailed and systematic discussion on the development of sensitized‐photocathode, from molecular dyes to quantum dots. Due to their intrinsic advantages in light response, exciton generation, and charge separation, semiconducting QDs sensitized photocathodes have shown apparent advances in efficiency and stability of PEC H_2_ evolution compared with organic dye counterparts. Specifically, we emphasize the vital importance of efficient hole transfer at the electrode interface in affecting the efficiency and durability of PEC systems. Finally, the challenges and expectations of sensitized‐photocathodes for water splitting in the future are addressed.

## 2 Overview of Photocatalytic and Photo‐Electrochemical Water Splitting

### 2.1 Photocatalytic Water Splitting

**Figure 1:**
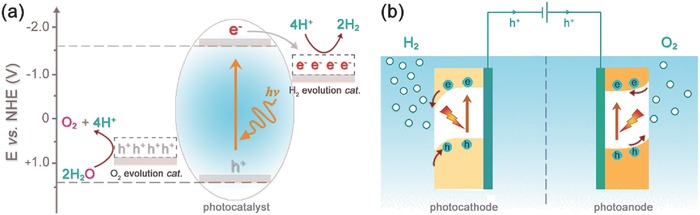
Schematic illustration of the two main models for artificial photosynthesis: a) photocatalytic water splitting in water; b) photo‐electrochemical water‐splitting system.

In a photocatalytic/photochemical water‐splitting system, as shown in **Figure** **1**a, at least one kind of light absorber, including semiconductor materials or molecular counterparts, is involved and suspended in aqueous solution. When irradiated with solar energy, light absorbers can be excited to produce photogenerated excitons. Taking semiconducting materials as an example, the photogenerated charges of electron and hole can migrate to H_2_ evolution cocatalysts and O_2_ evolution cocatalysts, and then react with water to produce H_2_ and O_2_, respectively.^31^, ^32^, ^33^ Afterwards, the excited semiconductors will go back to ground state and absorb another incident photon, leading to the consecutive production of molecular H_2_ and O_2_.^34^, ^35^, ^36^, ^37^, ^38^, ^39^ To realize overall water splitting, several principles are required to be satisfied. The first is the appropriate energy band position. Different from insulators or conductors, semiconductors own specific energy band structure, as shown in Figure 1a, giving a bandgap between the valence band (VB) and conduction band (CB).^40^ To realize overall water splitting, the conduction band edge needs to lie above the H^+^/H_2_ potential while the valence band edge must locate below the O_2_/H_2_O potential. Taking overpotentials involved in proton reduction and water oxidation into consideration, semiconductors are required to own a bandgap larger than ≈1.7 eV at room temperature for the purpose of overall water splitting.^41^ Because of the rigorous band energy requirement, only a few artificial systems can produce O_2_ and H_2_ simultaneously, while the efficiency is comparatively low and unsatisfactory for practical application.^42^, ^43^, ^44^, ^45^, ^46^ To solve this problem, an effective strategy is to separate the overall water splitting process into two half reactions, the O_2_ generation reaction and the H_2_ generation or CO_2_ reduction reaction.^47^, ^48^, ^49^, ^50^, ^51^, ^52^, ^53^ In both of the two half reactions, corresponding sacrificial redox agents, sacrificial electron donor or sacrificial electron acceptor, have to be added to provide the required electrons or oxidative equivalents. For concept study, sacrificial reagents involved systems are with vital importance to prove the principle. However, these sacrificial agents are always irreversible and expensive, which is contradictory to the ultimate goal of fabricating large‐scale and low‐cost solar assisted water‐splitting systems. Moreover, even those well‐designed Z‐scheme systems, combining with at least two kinds of semiconductors together, can realize the overall water splitting in the absence of sacrificial agents, the efficiency is still in a relatively low level and the resulting O_2_ and H_2_ cannot be separated automatically.^54^, ^55^, ^56^, ^57^, ^58^, ^59^ Thus, photocatalytic water splitting still faces many unsolved problems, including efficiency, device design, and costing, which limit its practical application.

### 2.2 Photo‐Electrochemical Water Splitting

Compared with photocatalytic systems, the integrated PEC water‐splitting counterparts can overcome the above‐mentioned disadvantages to a great extent. Typically, a conventional PEC system is composed of at least one photoactive semiconductor electrode, either n‐type or p‐type, as the working electrode.^60^, ^61^ The counter electrode, consisting of either metal (e.g., Pt) or semiconductor, is connected with a working electrode by external circuit. The combination of above two electrodes immersed in the electrolyte solution comprises the basis of a PEC cell (Figure 1b). And in general, a bias potential is needed in single‐photoelectrode PEC systems to further improve the efficiency of charge separation.^62^, ^63^, ^64^, ^65^

Furthermore, the contact between semiconductor electrode and electrolyte leads to a charge transfer process at the solid/liquid interface due to the difference of electrochemical potential between the two phases. Taking the photocathode, normally consisting of a p‐type semiconductor electrode, as an example (Figure 1b), when it is immersed into the electrolyte solution, the holes, as the majority charge carriers, will flow to the liquid phase until an equilibrium state is reached.^66^, ^67^, ^68^ This phenomenon results in excess positive charges in the solution while excess negative charges in the semiconductor electrode. As a result, space charge region between the interface of semiconductor electrode and electrolyte solution is established, which can offset the initial electrochemistry potential difference between semiconductor and solution. Due to above mentioned reason, band bending of semiconductor material, both cathodic and anodic electrodes, will occur near the interfacial region, as shown in Figure 1b.^69^, ^70^, ^71^

Under light irradiation, the p‐type semiconductor will absorb photons with energy equal to or exceeding its bandgap, thus generating the photoinduced charge carriers. To be specific, the electrons in VB will be excited to CB, the corresponding “hole” is left in its original site. Due to the existence of built‐in electric field and band bending, the photoexcited holes will move to the bulk of the p‐type semiconductor, while photoexcited electrons transport to the surface of semiconductor, followed by being injected into the electrolyte. Later, the holes transfer to the counter electrode (e.g., Pt) through the outer circuit and oxidize water to generate O_2_ while the electrons are used to reduce proton into molecular H_2_, simultaneously. In general, the p‐type semiconductors are used as photocathodes so that the minority carriers (electrons) can be directed to the solid/liquid interface for subsequent reduction reactions, especially H_2_ generation. Photoanodes, usually composed of n‐type semiconductors, operate in an inverse mode, which have been well‐discussed elsewhere.^72^

As discussed above, in a well‐designed PEC system, overall water splitting can be achieved in the absence of any sacrificial reagent. Moreover, photogenerated H_2_ and O_2_ are spatially separated. Still, similar to the photocatalytic water‐splitting systems in water, an ideal photoelectrode should satisfy the following criteria to realize efficient overall water splitting: (i) suitable band energy edge position for proton reduction and/or water oxidation; (ii) large scope and strong absorption in solar spectrum; (iii) high photochemical stability in aqueous electrolyte; and (iv) easy to obtain and low cost.^73^, ^74^ The currently developed photocathode materials can be divided into the following categories, metal oxide, Si, copper‐based chalcogenides, and III–V group materials. Among them, metal oxide semiconductors, such as NiO (3.5 eV), Cu_2_O (2.0 eV), CaFe_2_O_4_ (1.9 eV), and CuFeO_2_ (1.5 eV), often have advantages of easy preparation and low cost,^75^, ^76^, ^77^ though some of them suffer from low charge‐carrier mobility and short charge‐carrier lifetime, thus inhibiting their widespread application in PEC systems. Besides, p‐silicon demonstrates very high electronic mobility (up to 1360 cm^2^ V^−1^ s^−1^) and broad solar absorption spectrum, which make it an attractive photocathode material for artificial photosynthesis. However, the stability of silicon in aqueous solution is poor, which is a major obstacle to be solved in PEC water splitting system. Compositing p‐Si with other materials or cocatalysts is crucial and promising. The most commonly used III–V group materials include GaP (2.2 eV) and InP (1.34 eV), which are usually existing in the form of nanowire structure to reduce their cost and improve charge collection. In addition, heterojunction is also used to improve the PEC performance of such materials, which inevitably increases the complexity of the electrode construction to some extent. The copper‐based chalcogenides are another important branch as photocathode materials with favorable visible‐light response due to the narrow bandgaps, such as CuIn*_x_*Ga_1−_ *_x_*Se_2_ (1.68 eV), CuGaSe_2_ (1.7 eV), CuInS_2_ (1.5 eV), and so on.^78^ Some of them face the problems of poor stability and structural modification.

Clearly, almost no single material can meet all of the above‐mentioned demands simultaneously. So, numerous investigations have been focused on the design and fabrication of PEC water‐splitting systems in the past 40 years.^24^, ^79^, ^80^, ^81^, ^82^, ^83^, ^84^, ^85^, ^86^, ^87^, ^88^, ^89^, ^90^ However, there exists a tendency that researchers always pay their attention to the exploitation of photoanodes, such as titanium dioxide (TiO_2_), while often ignoring the development of efficient photocathodes.^91^ An analogous phenomenon also exists in the investigation of solar cell, mainly owing to the scarcity of suitable p‐type semiconductors.^92^, ^93^, ^94^

The focus on photoanode has made this field fast‐developing, while on the opposite, photocathode always develops sluggishly. So, a mismatch between photoanodes and photocathodes in both photocurrent density and catalytic efficiency emerges. The mismatching greatly hinders the construction of high‐efficient tandem PEC water‐splitting system, in the absence of extra bias between two photoelectrodes.^67^, ^95^, ^96^, ^97^, ^98^ Besides, for the photoanodes, self‐oxidation is easy to occur because of low transfer efficiency of photogenerated holes, which makes it difficult to use nonoxide materials. By contrast, the photocathode can avoid photooxidation to a great extent, because photoexcited holes can be transmitted to the external circuit before self‐oxidation happening due to the downward band bending at the solid/liquid interface and an applied external potential.^99^ As a result, the exploration of efficient photocathodes has not been restricted to metal oxide semiconductors. In addition, exploring photocathodes can help us understand the factors controlling the rate of hole photoinjection, which is beneficial for rational design of efficient photocathodes. Hence, establishing photocathodes with exceptional light absorption ability, high stability, and great conductivity is of great significance.^100^

## 3 Sensitized Photocathode for Water Splitting

Similar to photocatalytic water splitting process, PEC water‐splitting process can also be divided into three critical steps: (i) solar light harvesting; (ii) charge separation and charge diffusion; (iii) surface reduction/oxidation reaction. Considering the composition of solar spectrum, nearly half of the solar energy incident on the Earth's surface lies in visible‐light region (400 nm < λ < 800 nm). The ultraviolet light region, however, accounts for only 4% of the full spectrum.^101^, ^102^ So, it is very practically important to take full advantage of the visible light to improve the overall efficiency of PEC systems. Nevertheless, there always exists a dilemma between the light response scope and electrochemical reaction dynamics. It means that almost no semiconductor can own both adequately narrow bandgaps for exceptional light response and appropriate energy levels for catalytic reactions simultaneously, thus leading to either poor absorption performance or additional undesirable external bias. Therefore, the first step of photon absorption can be a big limiting factor, which is regarded as the foundation of establishing high‐efficient PEC systems.^103^, ^104^, ^105^, ^106^

**Figure 2:**
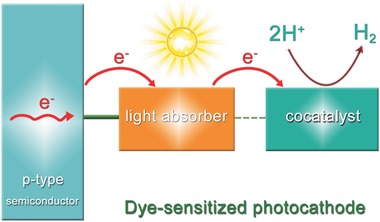
Main concept of the dye‐sensitized photocathode: consisting of p‐type semiconductor, light absorber, and cocatalyst.

Based on the above mentioned situations, various approaches have been utilized to coordinate the above‐mentioned two paradoxes.^107^, ^108^, ^109^ Inspired by the p‐type dye‐sensitized solar cells, the strategies of constructing dye‐sensitized photocathodes for H_2_ evolution in PEC systems have been intensively investigated.^110^, ^111^, ^112^, ^113^, ^114^, ^115^ In a common model, dye molecules are often attached onto the surface of p‐type semiconductors to work as a light absorber. Under visible‐light irradiation, molecular dyes can inject photo holes to p‐type semiconductor for water oxidation on counter electrode. At initial research stage, coordination compounds based on Ru, Os, and Ir metal centers have been widely employed as photosensitizers for their unique photophysical and electronic properties. However, the scarcity of these noble metals limits their large‐scale application. Due to this detrimental disadvantage, the development and utilization of low‐cost metal complexes, such as Mn, Fe, and Ni, have gradually become the hot spot of this field. Moreover, the free energy constraints also hinder the application of dye molecules alone. So, corresponding cocatalysts for H_2_ evolution are inevitable in a p‐type dye‐sensitized PEC system to accelerate carrier migration.^116^, ^117^, ^118^ With the coupling of light absorbers and cocatalysts, facile and favored interfacial charge migration can be achieved, leading to great advances in PEC H_2_ evolution of dye‐sensitized photocathode (**Figure** **2**). Up to now, the most investigated H_2_ evolution cocatalysts are cobalt‐based molecules, such as cobaloximes and diimine–dioxime cobalt complexes, which show high catalytic efficiency and favorable robustness.

### 3.1 Molecular Dye‐Sensitized Photocathode

**Figure 3:**
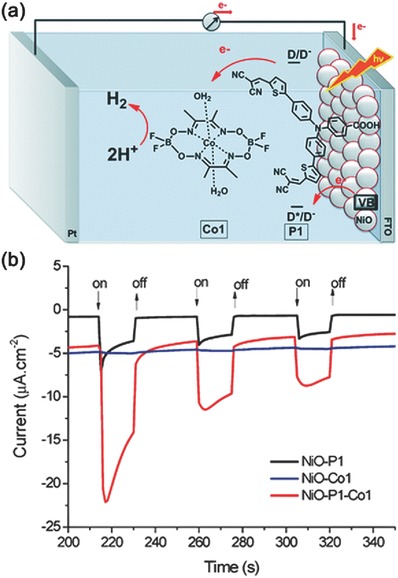
a) Schematic illustration of PEC device with a photocathode based on a P1‐sensitized NiO film on fluorine‐doped tin oxide (FTO) coated with Co1. b) The transient current responses to on–off cycles of illumination on photocathodes under an applied potential of 0.4 V versus Ag/AgCl in pH 7.0 phosphate buffer solution (λ > 400 nm). Reproduced with permission.^119^ Copyright 2014, Royal Society of Chemistry.

In 2012, Sun and co‐workers reported the first dye‐sensitized photocathode for PEC water splitting based on p‐type NiO.^119^ In this pioneer work, an organic molecular dye P1 and a cobaloxime complex catalyst Co1 for H_2_ evolution were integrated onto NiO to construct the photocathode (**Figure** **3**a). When tested in a typical three‐electrode PEC system in phosphate buffer solution (pH = 7), the system could generate a transient photocurrent density of ≈−20 µA cm^−2^ with an applied potential of −0.4 V (vs Ag/AgCl) (Figure 3b). Control experiments indicated that much lower photocurrent density could be detected in the absence of P1 or Co1, providing evidence on the electron transfer from P1 to Co1. A modified Clark‐type electrode sensor further confirmed the evolution of H_2_. However, due to exfoliation and/or decomposition of Co1 from the surface of NiO, the system suffered from poor stability.^120^ So, rational methods need to be developed to construct more robust photocathodes using molecular dyes.

**Figure 4:**
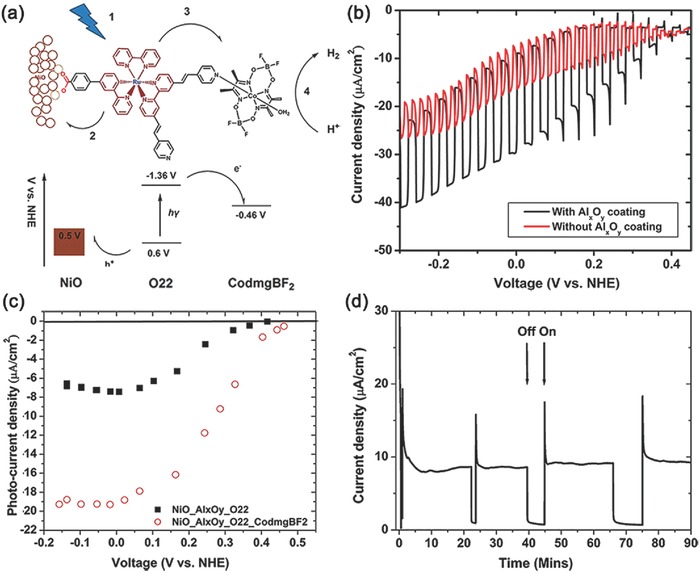
a) Scheme and energy diagram of the dye‐sensitized NiO electrode. b) linear sweep voltammetry (LSV) of NiO electrode with and without alumina coating. c) Plot of steady photocurrent versus applied bias for the O22‐sensitized NiO with (open circles) and without (solid squares) the CodmgBF2 catalyst. d) Chronoamperogram of the alumina coated NiO electrode loaded with O22 and CodmgBF2 catalyst at an applied bias of 0.1 V versus NHE. Reproduced with permission.^121^ Copyright 2013, American Chemical Society.

One great breakthrough was made by Wu and co‐workers in 2013.^121^ By using a supramolecular approach, a bifunctional ruthenium sensitizer O22 could coordinate with a cobaloxime catalyst (CodmgBF2) through its free pyridine units, resulting in the formation of a supramolecular assembly (**Figure** **4**a). Furthermore, the assembly could be firmly anchored onto the surface of NiO by carboxyl acid group. To further prevent the undesirable charge recombination between NiO film and sensitizer, a monolayer of alumina was introduced onto the surface of the electrode by atomic layer deposition, leading to an enhanced photocurrent (Figure 4b).^122^ A more positive onset potential could be observed in the presence of molecular catalyst, likely due to the faster electron transfer from sensitizer to the catalyst (Figure 4c). In a typical three‐electrode PEC system, the resultant supramolecular photocathode exhibited commendable stability to give a current density of about −9 µA cm^−2^ (0.1 V vs normal hydrogen electrode (NHE), phosphate buffer solution of pH 7) (Figure 4d). During a 2.5 h photoelectrolysis, the Faradaic efficiency of H_2_ evolution was about 68%. Such a smart design was feasible for efficient charge transfer because of the close proximity between cocatalyst and dye molecules, meanwhile spatial separation of cocatalyst and NiO prevented undesired charge recombination. Clearly, this work provides a model of using supramolecular method to construct dye‐sensitized photocathodes with improved stability of H_2_ evolution.^123^

**Figure 5:**
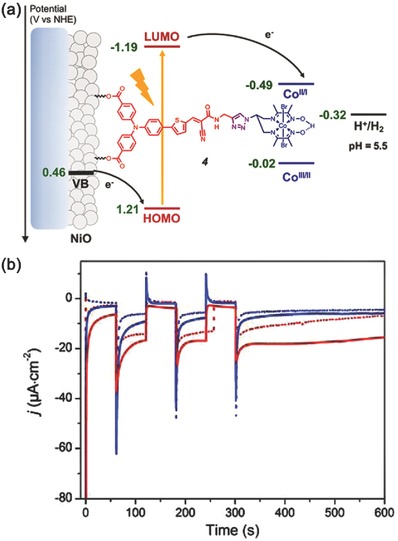
a) Energy diagram and working principle of the p‐type photocathode based on **4** at pH 5.5. b) Chopped‐light chronoamperometric measurements at **4**‐sensitized NiO electrodes poised at 0.14 (red traces) and 0.54 V (blue traces) versus RHE in the absence (dotted) and in the presence of chenodeoxycholic acid (CDCA) (plain) under visible‐light irradiation (400–800 nm, 65 mW cm^−2^, 1 sun) in pH 5.5 2‐(N‐morpholino) ethanesulfonic acid (MES) 0.1 m/NaCl 0.1 m supporting electrolyte. Reproduced with permission.^124^ Copyright 2016, American Chemical Society.

Subsequently, Artero and co‐workers further developed the concept of supramolecular assembly in fabricating photocathode and reported the first noble‐metal free and covalent dye‐catalyst dyad photocathode (**Figure** **5**a).^124^ The resulting photocathode could work in mildly acidic aqueous condition to give a photocurrent density about −15 µA cm^−2^ at an external bias of 0.14 V versus reversible hydrogen electrode (RHE). The whole system showed excellent stability while giving a fairly low Faradaic efficiency of 8%–10% for H_2_ evolution (Figure 5b), which was attributed to the competitive reduction of bulk NiO film evidenced by postmortem X‐ray photoelectron spectroscopy analysis. The covalent linking provides a much tighter interaction between sensitizers and cocatalysts, leading to a more stable system. While concerning its unsatisfactory efficiency and complicated fabrication process, more efforts should be devoted to establishing advanced photocathodes.^125^, ^126^, ^127^

**Figure 6:**
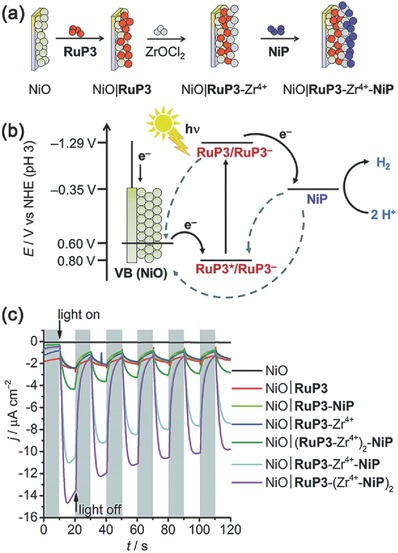
a) Assembly of supramoleular dye‐catalyst on photocathode: layer‐by‐layer deposition of RuP3, Zr^4+^, and NiP on p‐type NiO. b) Energy diagram of the p‐type photocathode. c) Chronoamperometry of NiO working electrodes under chopped light irradiation at *E*_appl_ = 0.3 V versus RHE. All experiments were performed in a 3‐electrode setup with Ag/AgCl/KCl (sat.) reference and Pt mesh counter electrodes. Reproduced with permission.^117^ Copyright 2016, Royal Society of Chemistry.

In addition to the coordination and covalent interactions to achieve dye–catalyst assembly, some inorganic metal ions can also make similar effects.^117^ By using a layer‐by‐layer deposition approach, Reisner and co‐workers developed a dye‐sensitized NiO photocathode with a hexaphosphonated Ru(2,2′‐bipyridine)_3_ based dye (RuP3) and a [Ni(P_2_N_2_)_2_]^2+^ type proton reduction catalyst (NiP) (**Figure** **6**a). Zr^4+^ ions were used as the linkage between RuP3 dye and NiP catalyst, resulting in a supramolecular assembly on NiO nanoparticles. Figure 6b illustrated the involved interfacial charge transfer processes of this system under visible‐light irradiation. The electrode showed much higher photocurrents and better stability than photocathode with RuP3 and NiP co‐immobilised on NiO surface without Zr^4+^ cations (Figure 6c). However, the corresponding Faradaic efficiency of H_2_ evolution was only about 8.6 ± 2.3%, which was attributed to the fast recombination reactions at the interface between reduced NiP and oxidized RuP3 or NiO. Anyway, the use of inorganic ions to construct sensitized photocathodes demonstrates potential for PEC water splitting.

**Figure 7:**
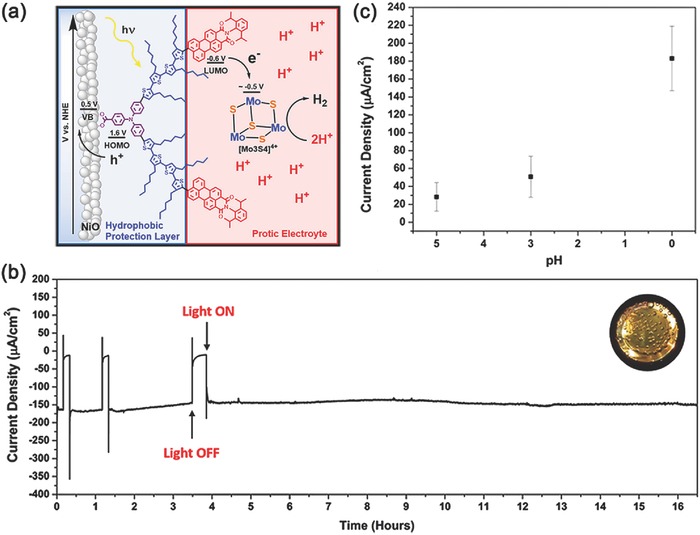
a) Molecular structure of BH4 and the schematic showing the energetics of the dye‐sensitized photo‐electrochemical cell (DSPEC). b) Chronoamperometry of a BH4‐sensitized NiO film in pH 0 (1 m HCl) solution with 5 × 10^−3^ m [Mo_3_S_4_]^4+^ catalyst at an applied potential of 0 V with constant light illumination (aside from 3 light on/off tests). Inset: H_2_ bubbles formed on the working electrode. c) Current densities of a BH4‐sensitized NiO film in pH 5, 3, and 0 solutions, all containing 5 × 10^−3^ m [Mo_3_S_4_]^4+^ catalyst. Reproduced with permission.^129^ Copyright 2016, American Chemical Society.

On the basis of the above‐mentioned works, it can be found that, at present stage, dye‐sensitized photocathodes are always limited by poor stability and/or low catalytic efficiency.^128^ To solve these problems, Wu and co‐workers reported an acidically stable p‐type dye‐sensitized PEC system based on a NiO film (**Figure** **7**a).^129^ In their system, a bifunctional organic donor–acceptor dye (BH4) was used to realize the striking stability of the photocathode, which was inspired by the hydrophobic/hydrophilic properties of lipid bilayer membranes. On one hand, the photosensitizer harvests sunlight efficiently and anchors onto the surface of NiO firmly; one the other hand, its hydrophobic π linker can protect the semiconductor surface from protons and water.^130^ With the incorporation of a non‐noble‐metal cluster (Mo*_x_*S*_y_*) as the cocatalyst, this system produced a proton‐reducing current of −183 ± 36 µA cm^−2^ at pH 0 (0 V vs NHE). Strikingly, this current value could sustain more than 16 h without obvious decline (Figure 7b). The impressive durability of PEC H_2_ evolution has never been shown before for any dye‐sensitized PEC systems, either n‐type or p‐type. Unfortunately, the current density was heavily dependent on pH values of the electrolyte, as shown in Figure 7c. Only under extremely acidic conditions, this kind of sensitized photocathode could show such a superb performance of H_2_ evolution, which might restrict its practical utilization. Herein, more powerful methods are still desired for making sensitized photocathodes proceeding effectively in mild, pragmatic, and ideal conditions.^131^

### 3.2 Quantum Dots Sensitized Photocathode

**Scheme 2:**
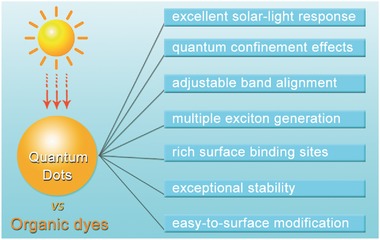
Intrinsic advantages of semiconducting quantum dots versus organic dyes working as light absorbers.

Up to now, though great efforts have been devoted to developing efficient dye‐sensitized photocathodes for PEC water splitting, the poor stability and low current density, mainly due to the intrinsic properties of organic molecule dyes, severely restrict their further practical applications, see detailed summary in **Table** **1**. Employing novel chromophores with better light response and stability is in great necessity. Compared with organic dye molecules, semiconductor QDs, such as CdSe QDs, CdS QDs, CdTe QDs, etc., possess plenty of superiorities in terms of absorptivity, tunability, and durability, thus considered as a promising alternative for PEC water‐splitting application, see details in **Scheme** **2**.^132^, ^133^, ^134^, ^135^, ^136^, ^137^, ^138^, ^139^, ^140^ First, the absorption coefficients of QDs are comparatively higher toward organic dyes. Second, because of quantum confinement effects, QDs possess size‐dependent absorption properties. It means that, by controlling the size, we can further enhance their exploitation of solar spectrum.^141^, ^142^, ^143^, ^144^, ^145^, ^146^, ^147^, ^148^ Third, the band positions of QDs can be easily modulated, which affords potential advantages for accelerating photoinduced charge separation and migration. Fourth, multiple exciton generation by a single photon is another important characteristic of semiconductor QDs, which can meet the demands of multiple‐charge redox reactions, such as photocatalytic H_2_ production. Moreover, rich surface binding sites and easy‐to‐surface modification make QDs ready to assemble with electrode and cocatalysts.^149^ These privileges make QDs as promising and versatile sensitizers for fabricating high‐efficient and stable PEC systems.^150^, ^151^, ^152^, ^153^, ^154^ **Table** **2** gave a detailed summary of the currently reported examples of QDs‐sensitized photocathodes for H_2_ evolution.

**Table 1:** Detailed summary of the reported dye‐sensitized photocathodes up to date

Photocathode	Electrolyte/pH	Bias	Current density	Stability	Faradaic efficiency [%]
NiO/P1/Co1^119^	0.1 m phosphate pH 7 buffer solution	−0.4 V versus Ag/AgCl	−20 µA cm^−2^	<10 min	–
NiO/PMI‐6T‐TPA^95^	0.1 m Na_2_SO_4_ pH 7 solution	0 V versus Ag/AgCl	−2.2 µA cm^−2^	4 h	98
NiO/O22/Co(dmg)BF_2_ 121	0.1 m phosphate pH 7 buffer solution	0.1 V versus NHE	−10 µA cm^−2^	2.5 h	68
NiO/RuP/CoHEC^96^	0.1 m phosphate pH 7 buffer solution	−0.4 V versus Ag/AgCl	−13 µA cm^−2^	–	–
C/polyRu/MoS*_x_* 112	0.5 m H_2_SO_4_ pH 0.3 solution	0.1 V versus Ag/AgCl	−15 µA cm^−2^	2 h	98
ITO/PEDOT:PSS/P3HT:C60–Co^187^	0.1 m acetate buffer pH 4.5	0.1 V versus NHE	−3 µA cm^−2^	1 h	97
NiO/343 dye/[FeFe]‐ hydrogenase mimic^188^	0.1 m acetate buffer pH 4.5	−0.3 V versus Ag/AgCl	−10 µA cm^−2^	1100 s	50
NiO/RuP/Rh^123^	CH_3_CN, 0.1 m [Bu_4_N]ClO_4_ solution	−0.45 V versus Ag/AgNO_3_	−80 µA cm^−2^	4 h	85
NiO/BH4/MoS*_x_* 129	1 m HCl pH 0	0 V versus NHE	− 183 ± 36 µA cm^−2^	16 h	49 ± 11
NiO/4/Co^124^	pH 5.5 MES 0.1 m/NaCl 0.1 m	0.14 V versus RHE	−15 µA cm^−2^	3 h	8–10
NiO/RuP3/Zr^4+^/NiP^117^	0.1 m Na_2_SO_4_ pH 3 solution	0.3 V versus RHE	−9.97 µA cm^−2^	2 h	8.6 ± 2.3
NiO/Ru/Co^189^	0.1 m phosphate pH 7 buffer solution	0.2 V versus NHE	−60 µA cm^−2^	1 h	–
NanoITO/‐DA‐Zr‐RuP2^2+^‐Zr‐NiP^2+^ 107	0.1 m MES buffer pH 5.1	0.25 V versus NHE	−56 µA cm^−2^	4 h	53 ± 5.2
NiO/PMI‐6T‐TPA/Pt_ed_ 128	0.1 m H_2_SO_4_	0.059 V versus RHE	−30 µA cm^−2^	10 h	100
NiO/PMI/30ALD/CoL_2_ 190	0.1 m H_2_SO_4_ and 0.1 m Na_2_SO_4_ in 1:1 H_2_O:MeCN	−0.40 V versus Ag/AgNO_3_	−25 µA cm^−2^	2 h	60 ± 10
NiO/1/Co^191^	0.1 m acetate buffer pH 4.5	0 V versus Ag/AgCl	−270 µA cm^−2^	–	–

**Table 2:** A detailed summary of the reported QDs‐sensitized photocathodes up to date

Photocathode	Electrolyte/pH	Bias	Current density	Stability	Faradaic efficiency [%]
Au/InP QDs/[Fe_2_S_2_(CO)_6_]^155^	0.1 m NaBF_4_ pH 7 solution	−0.4 V versus Ag/AgCl	−100 nA cm^−2^	1 h	60
Au/P3HT/CdSe QDs/Pt^152^	0.1 m phosphate pH 7 buffer solution	−0 V versus RHE	−1.2 mA cm^−2^	–	80–100
p‐Si/InP QDs/[Fe_2_S_2_(CO)_6_]^157^	0.1 m H_2_SO_4_	−0.5 V versus Ag/AgCl	−1.2 mA cm^−2^	40 min	–
NiO/CdSe QDs/NiS^161^	0.5 m Na_2_SO_4_ pH 6.8 solution	0 V versus Ag/AgCl	−130 µA cm^−2^	1.5 h	95
NiO/CdSe QDs/[Co(bdt)_2_]^−^ 162	0.1 m KCl pH 7 solution	−0.28 V versus RHE	−2 mA cm^−2^	18 h	100 ± 2
NiO/CdSe QDs^164^	0.1 m Na_2_SO_4_ pH 6.8 solution	−0.1 V versus NHE	−60 µA cm^−2^	45 h	≈100
NiO/CdSe QDs/CoHEC^168^	0.1 m Na_2_SO_4_ pH 6.8 solution	0 V versus NHE	−110 µA cm^−2^	3.5 h	≈81
Au/SWCNT/InP/ZnS QDs^170^	0.1 m phosphate pH 7 buffer solution	0 V versus Ag/AgCl	−0.7 µA cm^−2^	1 h	–
Cu_2_S/C QDs^171^	1 m KCl pH 5.97 solution	0 V versus NHE	−1.05 mA cm^−2^	0.5 h	–
NiO/CdTe QDs/NiS^172^	0.1 m phosphate pH 6 buffer solution	−0.222 V versus Ag/AgCl	−40 µA cm^−2^	8.5 h	≈100
NiO/CdSe QDs^173^	0.1 m phosphate pH 6.8 buffer solution	0 V versus RHE	−20 µA cm^−2^	–	–
NiO/CdSe QDs/MoS_2_ 174	A buffer solution (pH 6) containing 0.3 m C_6_H_12_N_4_, 0.1 m HCl, and 0.2 m KCl	−0.131 V versus RHE	−60 µA cm^−2^	3.5 h	≈100
NiO/PTZ/CdSe QDs^183^	0.1 m Na_2_SO_4_ solution	−0.1 V versus NHE	−180 µA cm^−2^	20 h	≈100
GDY/CdSe QDs^186^	0.1 m Na_2_SO_4_ solution	0 V versus NHE	−70 µA cm^−2^	4 h	90 ± 5
NiO/CdSe QDs/CoHEC^192^	0.2 m HMTA/HCl pH 6 buffer solution with 0.1 m KCl	0 V versus Ag/AgCl	−115 µA cm^−2^	5 h	99.5
NiO/MAA/CdSe QDs^193^	0.1 m Na_2_SO_4_ pH 6.8 solution	−0.1 V versus NHE	−136 µA cm^−2^	30 h	91

**Figure 8:**
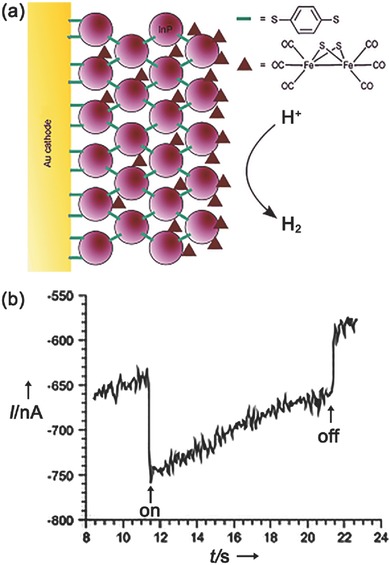
a) Cross‐section of a InP QD‐modified gold electrode with adsorbed/intercalated [Fe_2_S_2_(CO)_6_] subsite analogue. b) Photocurrent measured at a bias potential of 400 mV versus Ag/AgCl in 0.1 m NaBF_4_ under illumination with a 395 nm light‐emitting diode (LED). Reproduced with permission.^155^ Copyright 2011, Wiley‐VCH.

The first quantum dot‐sensitized photocathode for PEC H_2_ evolution was reported by Pickett and co‐workers.^155^ In the pioneer work, presynthesized InP QDs were assembled onto the surface of a gold electrode through a layer‐by‐layer method, as shown in **Figure** **8**a. Then, [FeFe]‐hydrogenase mimics, [Fe_2_S_2_(CO)_6_], were introduced onto the Au/InP QDs photoelectrode by coordination through their sulfide bridges with QD surface sites.^156^ Under light irradiation, a nanoampere‐level photocurrent was generated during 1 h experiment at a bias potential of 400 mV versus Ag/AgCl electrode (Figure 8b). Gas‐chromatography analysis further verified the evolution of H_2_ gas with a Faradaic efficiency of ≈60%. Although the efficiency is beyond the realm of practical application, this simple PEC system lays the foundation of constructing sensitized photocathodes using QDs. Moreover, the robustness of the system, a fatal problem in organic dye‐sensitized PEC counterparts, shows us the potential of utilizing QDs‐sensitized photocathode in PEC water splitting.

**Figure 9:**
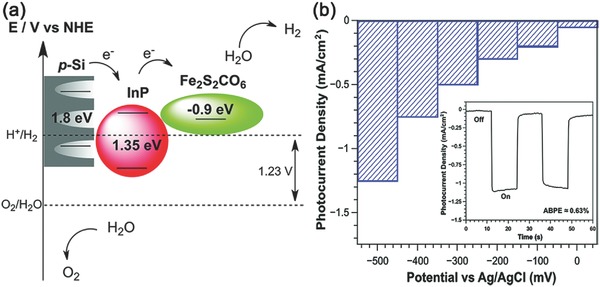
a) Schematic representation of the bandgap energy of InP QDs and Fe_2_S_2_(CO)_6_ catalyst attached inside p‐type Si as well as charge transfers leading to electrochemical reduction of protons to H_2_. b) The increase in photocurrent density with increasing bias potentials in steps of 100 mV for electrografted p‐Si loaded with InP and Fe_2_S_2_(CO)_6_ catalyst. The inset shows two light‐dark photocurrent cycles measured at a bias potential of 500 mV. Reproduced with permission.^157^ Copyright 2014, Royal Society of Chemistry.

Later, Nann and co‐workers reported another InP QDs sensitized porous silicon (p‐Si) photocathode for H_2_ evolution, which also used [FeFe]‐hydrogenase mimics as cocatalysts for proton reduction (**Figure** **9**a).^157^ After an electrochemical etching procedure, the resulting p‐Si not only owned a suitable bandgap of about 1.8–2.2 eV for visible‐light response, but also provided large surface area for loading QDs.^158^, ^159^ Taking 0.1 m H_2_SO_4_ as the electrolyte, the obtained photocathode demonstrated enhanced photocurrent density with external bias ramping up (Figure 9b). Under a bias potential of −0.5 V (vs Ag/AgCl), a photocurrent density of −1.2 mA cm^−2^ was observed, much higher than those without either InP QDs or Fe_2_S_2_(CO)_6_. Although the instability in aqueous solution and high cost of such materials restrict their further practical application, the importance of modification with hole transfer layer to enhance H_2_ production activity has been definitely revealed.

**Figure 10:**
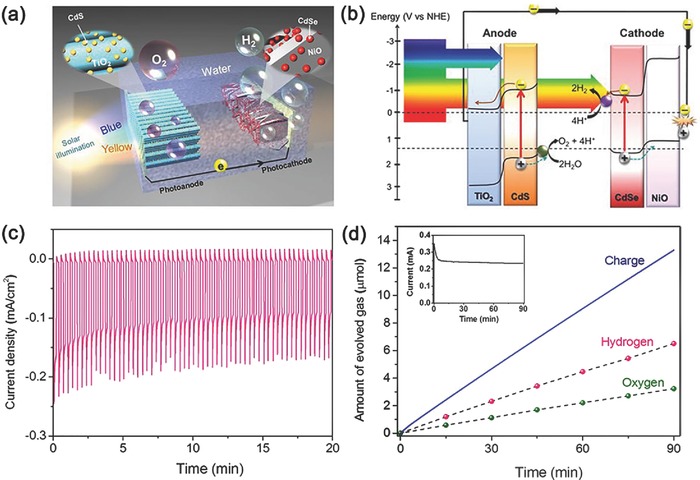
a) Cadmium chalcogenide QD‐modified photo‐electrochemical cell constructed with an array of CdS QDs modified TiO_2_ nanorods as photoanode and CdSe QDs modified NiO nanosheets as photocathode. b) Energy band diagram of a cadmium chalcogenide QD‐modified photo‐electrolysis cell. c) Photocurrent density versus time under chopped light exposure. d) Comparison of the evolved H_2_ and O_2_ gases and the charge through the external circuit. Inset panel in (d) shows the photocurrent versus time measurement. Reproduced with permission.^161^ Copyright 2014, American Chemical Society.

Compared with Au and silicon, p‐type NiO shows great privileges in PEC water splitting for its low cost, visible‐light transparency, and ability of hole transport.^160^ In 2014, Liu and co‐workers reported an unassisted tandem PEC water‐splitting system consisting of a CdSe QDs sensitized NiO photocathode and a CdS QDs sensitized TiO_2_ photoanode (**Figure** **10**a).^161^ Under visible‐light irradiation, both CdSe QDs and CdS QDs could be excited to generate electrons and holes. Then, photogenerated holes in CdS QDs transferred to IrO*_x_* for water oxidation and the photogenerated electrons in CdSe QDs reacted with proton to generate H_2_ taking NiS as cocatalyst. Under the optimal conditions, a short‐circuit current density of ≈0.125 mA cm^−2^ could be observed with no external bias potential and open‐circuit voltage (*V*_oc_) was ≈0.53 V (Figure 10b). The prolonged testing was performed under short‐circuit conditions and a stable photocurrent density of 0.11 mA cm^−2^ was obtained in 20 min (Figure 10c). A stoichiometric amount of H_2_ and O_2_ could be detected and the Faradaic efficiency was about 95% (Figure 10d). This work shows PEC water splitting a promising avenue for solar‐to‐fuel conversion. However, compared with well‐developed photoanode, the performance of photocathodes has become a major limitation for better solar water splitting. Herein, fabrication of high‐efficient and ultrastable QDs‐based photocathodes from a more facile pathway is in its great necessity.

**Figure 11:**
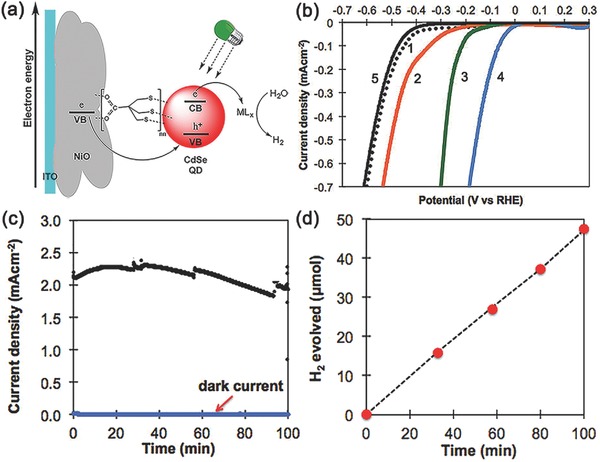
a) Scheme for electron transfer processes occurring in CdSe quantum dot‐sensitized photocathode in the presence of cocatalyst. b) Linear sweep voltammograms under 520 nm LED light (150 mW) illumination of (1) bare NiO film, (2) NiO‐S_3_‐cap‐CdSe, (3) NiO‐S_3_‐cap‐CdSe with Ni(DHLA)*_x_*, (4) NiO‐S_3_‐cap‐CdSe with [Co(bdt)_2_]^−^, and NiO‐S_3_‐cap‐CdSe without illumination at 0.1 m KCl solution (pH = 6). c) Controlled potential bulk photo‐electrolysis experiments for NiO‐S_3_‐cap‐CdSe/[Co(bdt)_2_] under light and dark conditions. d) Experimentally observed (red circles) and theoretically calculated (black dashed line) H_2_ production versus time for NiO‐S_3_‐cap‐CdSe with [Co(bdt)_2_]^−^ (1.85 × 10^−4^ m) at an applied potential of ‐0.28 V versus RHE. Reproduced with permission.^162^ Copyright 2014, American Chemical Society.

In 2015, Eisenberg and co‐workers reported a QD‐sensitized photocathode for PEC H_2_ evolution.^162^ As shown in **Figure** **11**a, water‐soluble CdSe QDs were anchored onto NiO through a molecular linker 3‐mercapto‐2,2‐bis(mercaptomethyl)propanoate (denoted as S_3_‐cap). Either [Co(bdt)_2_]^−^ (bdt = 1,2‐benzenedithiolate) or Ni(DHLA)*_x_* (DHLA = dihydrolipoic acid anion) complex was used as the H_2_‐forming catalysts.^163^ Four different PEC systems, including bare NiO film, NiO‐S_3_‐cap‐CdSe, NiO‐S_3_‐cap‐CdSe with Ni‐(DHLA)*_x_* and NiO‐S_3_‐cap‐CdSe with [Co(bdt)_2_]^−^, were tested to explore the differences of the onset potential (Figure 11b). In the presence of cocatalysts, positive shifts of nearly 100 and 200 mV relative to NiO‐S_3_‐cap‐CdSe electrode could be observed, indicating that addition of metal complexes in solution could significantly enhance H_2_ formation performance. With a bias of −0.28 V versus RHE in 0.1 m KCl, the NiO‐S_3_‐cap‐CdSe/[Co(bdt)_2_]^−^ photocathode achieved a current density of about −2 mA cm^−2^ (Figure 11c). H_2_ evolution was detected by gas chromatographic analysis to give a Faradaic efficiency of 100 ± 2% (Figure 11d). However, in view of the existence of large amount of Cl^−^, it preferred to oxidize Cl^−^ ions instead of water here.

**Figure 12:**
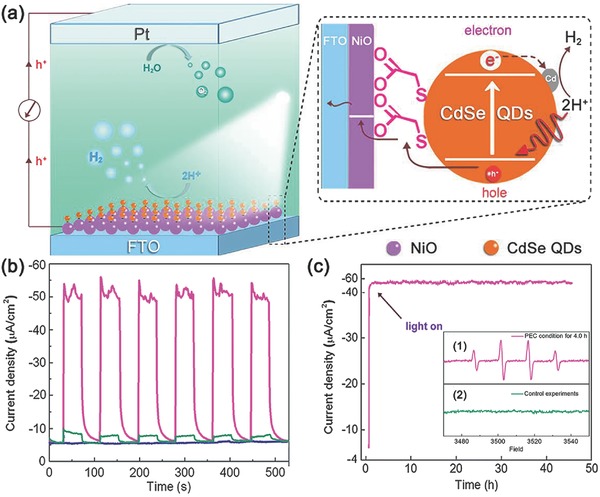
a) Illustration of the assembled photocathode in a PEC setup and the migration of photogenerated excitons at the interfaces. b) The transient photocurrent responses to on–off illumination of the NiO electrode (blue line), the electrode made via physical adsorption of CdSe QDs on NiO (dark cyan line), and the linker‐engineered CdSe QDs/NiO electrode (pink line). c) *J–t* curve of the assembled CdSe QDs/NiO electrode in a 45 h test. Reproduced with permission.^164^ Copyright 2015, Royal Society of Chemistry.

Simultaneously, Wu and co‐workers designed a PEC system based on QD‐sensitized NiO photocathode to achieve efficient and ultrastable solar H_2_ evolution, in which a molecular linker, mercaptoacetic acid (MAA), was chosen to construct a robust NiO/MAA/CdSe QDs photocathode for H_2_ evolution in neutral water (**Figure** **12**a).^164^ Under visible light, a photocurrent density of about −60 µA cm^−2^ at a bias of −0.1 V versus NHE was observed (Figure 12b) and no distinct current decline occurred within 45 h (Figure 12c). The extremely high performance of H_2_ evolution was attributed to the rational integration of CdSe QDs and NiO by a molecular hole relay, MAA, which played crucial roles in maintaining the charge balance of CdSe QDs for both electron and hole transfer processes at the nanoscale interfaces.^165^ The corresponding Faradaic efficiency for H_2_ evolution was nearly 100%. More importantly, electron paramagnetic resonance (EPR) experiment revealed the generation of •OH radicals on the anodic electrode, which was considered as necessary products for water electrolysis into O_2_.^166^ That is, an efficient and stable CdSe QDs/NiO photocathode was established, which could realize whole water splitting without any sacrificial reagent, external cocatalyst, protecting layer, and buffer solution. The amazing durability and near unity Faradaic efficiency of H_2_ production in this work indicate the great potential of using QDs in PEC water splitting application. Recent results indicated that assembling cocatalysts of [FeFe]‐hydrogenase mimics onto QD surface or modification the morphology of NiO layer to give a porous structure, the photocurrent density could be further improved due to the boosted processes of photoelectrons capture, hole migration, and proton reduction.^167^, ^193^

**Figure 13:**
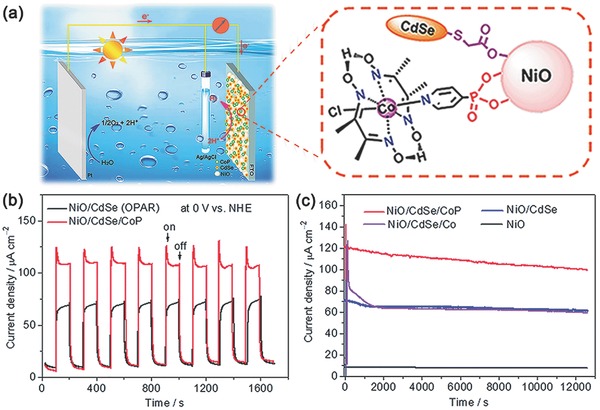
a) Schematic diagram of a photo‐electrochemical cell, consisting of a photocathode with a cobaloxime catalyst anchored to the CdSe QD‐sensitized NiO film on a FTO glass substrate, a Ag/AgCl reference electrode, and a Pt wire as counter electrode for water splitting. b) Transient current responses of the photocathodes made using the one‐pot adsorption and reaction (OPAR) procedure to the on–off cycles of illumination at 0 V versus NHE in a three‐electrode PEC cell in 0.1 m Na_2_SO_4_ solution (pH 6.8). c) Curves of photocurrent density versus time for the prepared porous NiO‐based photocathodes over 3.5 h illumination at an applied potential of 0.2 V. Reproduced with permission.^168^ Copyright 2015, Royal Society of Chemistry.

Later, Sun and co‐workers also reported a NiO‐based photocathode sensitized by CdSe QDs for H_2_ evolution.^168^ To accelerate the process of interfacial electron transfer, a cobalt complex catalyst, [CoCl(dmgH)_2_(pyridyl‐4‐hydrophosphonate)] (CoP, dmgH‐4‐dimethylglyoximate), was simultaneously and chemically adsorbed onto the surface of NiO (**Figure** **13**a). The results showed that the morphology of NiO had a larger effect on photocurrent than the loading forms of QDs.^169^ When open porous NiO electrode prepared by one‐pot adsorption and reaction procedures was used to fabricate photocathode, the resulting system could produce a photocurrent density of −110 µA cm^−2^ (0 V vs NHE). About 83% of the current density remained after 3.5 h illumination to give a Faradaic efficiency for PEC H_2_ evolution of ≈81%. Conversely, a reference electrode with physically adsorbed cobaloxime catalyst, denoted as NiO/CdSe/Co, showed poor stability (Figure 13c). Compared with those dye‐sensitized NiO photocathodes with analogous cobaloxime catalysts under similar conditions, this QD‐sensitized electrode exhibited better performance in both photocurrent density and stability. Control experiments manifested that such an excellent performance originated from broad visible‐light absorption of CdSe QDs and the existence of cobalt cocatalyst.

**Figure 14:**
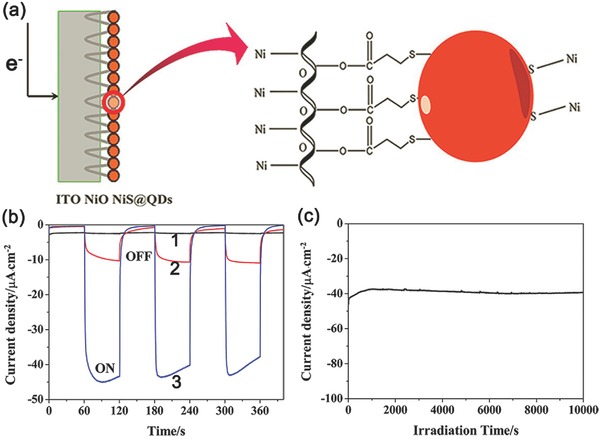
a) Scheme of the NiO/NiS/QDs photocathode. b) Photocurrent density response to on–off cycles under light illumination at −0.2 V versus Ag/AgCl in a pH = 6.0 buffer solution. c) Current–time curves of NiO/QDs photocathode under light illumination at −0.3 V versus Ag/AgCl in air saturated electrolyte (0.1 m KCl). Reproduced with permission.^172^ Copyright 2015, American Chemical Society.

Besides, other kinds of chalcogenide semiconductor QDs, such as CdTe QDs and CdS QDs, can also be used as photosensitizers in fabricating sensitized‐photocathode PEC systems.^170^, ^171^ In 2015, Dong et al. reported a CdTe QDs‐sensitized NiO photocathode for PEC water splitting (**Figure** **14**a).^172^ As shown in Figure 14b, in the absence of CdTe QDs, negligible photocurrent could be observed during the PEC measurement. When CdTe QDs were introduced, photocurrent increased slightly and a ≈32.5% Faradaic efficiency for H_2_ evolution could be obtained. Then, with the introduction of Ni^2+^ in the electrolyte, the above electrode showed an enhanced photocurrent density of about −45 µA cm^−2^. More importantly, the amount of H_2_ production was nearly 13 times than the situation without NiCl_2_, indicating that both QDs and cocatalysts were necessary for efficient H_2_ evolution. During a 30 000 s measurement, the photocathode showed good activity and stability for photo‐electrocatalytic H_2_ evolution, giving a Faradaic efficiency of nearly 100% (Figure 14c). The formation of NiS composition at the surface of QDs was found after a long‐time PEC experiment, which was considered as the actual active site for H_2_ evolution. The in situ formed NiS species could capture photogenerated electrons rapidly and lead to the enhanced activity of H_2_ generation. Similarly, others also reported a series of QDs‐sensitized photocathodes with various H_2_ evolution cocatalysts to improve electron capture and proton reduction processes.^173^, ^174^ These works indicate the great potential of QDs as alternative sensitizers in PEC systems.

### 3.3 Modified Quantum Dots Sensitized Photocathode

As a multi‐carrier and multi‐interface involved catalytic process, various factors can influence the final efficiency of solar‐to‐fuel conversion. How to further increase the efficiency and durability of H_2_ evolution using QDs‐sensitized photocathodes needs deeper thinking. Since generated in pairs, it is believed that the migration and consumption of photogenerated electrons and holes should be taken into consideration simultaneously. Spectroscopic studies have revealed that electron transfer usually occurs smoothly, especially with the assistance of cocatalysts. However, its counterpart, the hole transfer process, is about two to four orders of magnitude slower than that of electrons.^175^, ^176^, ^177^, ^178^, ^179^ Herein, the efficiency of interfacial hole migration is regarded as the rate‐determining step for solar H_2_ evolution using QDs. In other words, quick and effective capture and remove of photogenerated holes may take one step further toward making water splitting commercially viable.^180^

**Figure 15:**
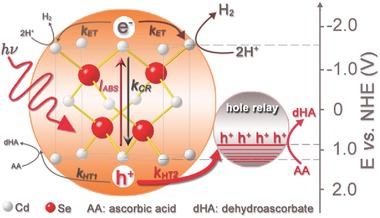
Schematic illustration of the solar H_2_ evolution of CdSe QDs in the presence and absence of hole‐accepting ligands (HAL) and the corresponding interfacial charge transfer processes. Reproduced with permission.^183^ Copyright 2015, Wiley‐VCH.

**Figure 16:**
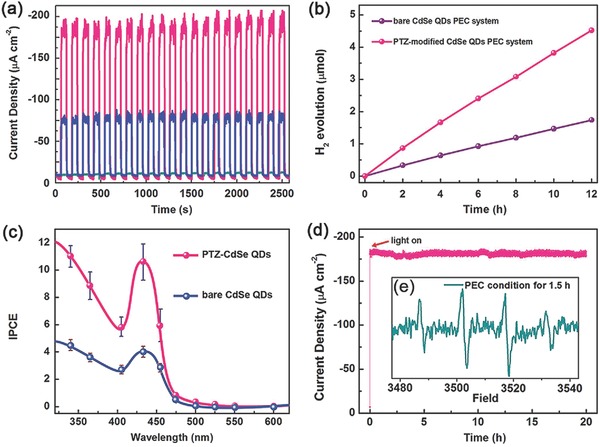
a) Transient photocurrent responses to chopped visible‐light irradiation of NiO electrode (cyan line), bare CdSe QDs electrode (blue line), and PTZ‐modified CdSe QDs electrode (pink line). b) IPCE spectra of bare CdSe QDs electrode (blue line) and PTZ‐modified CdSe QDs electrode (pink line). c) Time courses of H_2_ evolution of bare CdSe QDs electrode and PTZ‐modified CdSe QDs electrode as the working electrode, respectively. d) Long time *J*–*t* curve in 20.0 h test. Inset: EPR signal obtained from the three‐electrode PEC system by using 0.02 m 5,5‐dimethyl‐1‐pyrroline N‐oxide (DMPO) as a trapping agent under visible‐light irradiation (λ > 400 nm) and −0.1 V versus NHE for 1.5 h (e). Reproduced with permission.^183^ Copyright 2015, Wiley‐VCH.

With this in mind, Wu and co‐workers first employed hole‐accepting ligands to facilitate the process of interfacial hole capture and transfer (**Figure** **15**), which in turn dramatically increased the performance of solar H_2_ evolution of NiO/CdSe QDs photocathode. Phenothiazine (PTZ) was utilized as a hole accepting ligand to modify CdSe QDs for tight sulphur‐cadmium coordination,^181^, ^182^ inert visible‐light response, and suitable highest occupied molecular orbital level (≈0.9 V vs NHE) for hole capture and transfer. Herein, a NiO/PTZ/CdSe QDs photocathode through a simple solution‐processed method was prepared.^183^ Specifically speaking, a photocurrent density of about −180 µA cm^−2^ was observed when illuminated by a 300 W Xe‐lamp (λ > 400 nm) at −0.1 V versus NHE, which was 2.5‐fold to that of the photocathode without PTZ modification (**Figure** **16**a). During 12 h irradiation, H_2_ was evolved with a constant rate of 3000 µmol h^−1^ g^−1^ cm^−2^, with a Faradaic efficiency of nearly 100% for PEC H_2_ evolution (Figure 16b). In addition, the incident photon‐to‐current conversion efficiency (IPCE) plots of both electrodes matched well with the UV–vis absorption spectrum of CdSe QDs, and reached a maximum of ≈10.3% at 438 nm (Figure 16c). These results demonstrated the vital importance of PTZ to achieve efficient hole extraction and charge separation. The PEC system also showed favorable stability. No obvious current decay was observed during a long‐time measurement (20 h). As shown in Figure 16e, the high intensity of EPR signal verified that large amount of •OH radicals had been obtained through water oxidation by photogenerated holes. Obviously, this strategy of surface modification of QDs provided a guidance to overcome the hole‐transfer limitation and offered a new opportunity to further improve the efficiency of PEC H_2_ evolution.

**Figure 17:**
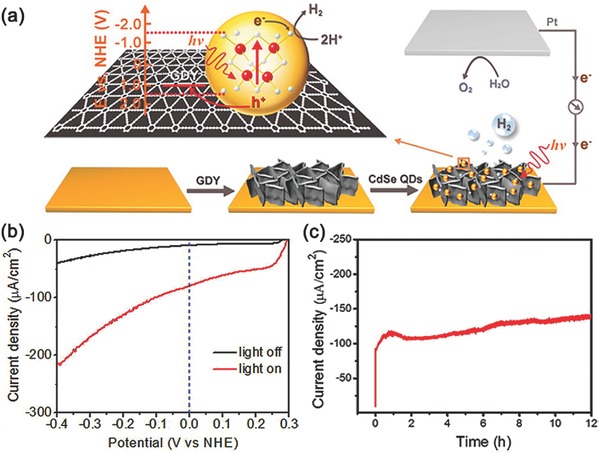
a) Schematic diagram of the PEC cell, consisting of the assembled CdSe QDs/GDY photocathode, Pt wire as counter electrode, and corresponding interfacial migration process of the photogenerated excitons. b) LSV scanning from 0.3 to −0.4 V at 2 mV s^−1^ with light off (black trace) and on (red trace) for CdSe QDs/GDY photocathode. c) Controlled potential electrolysis of CdSe QDs/GDY photocathode during 12 h test. Reproduced with permission.^186^ Copyright 2016, American Chemical Society.

Except the modification of QDs with hole‐accepting ligands, exploring and developing novel hole transfer layer for constructing QDs‐sensitized photocathode is another promising approach to extract and transfer photogenerated holes. Graphdiyne (GDY), a novel π‐conjugated carbon material, possesses fairly high hole mobility of 10^4^ cm^2^ V^−1^ s^−1^, as well as a conductivity of 2.516 × 10^−4^ S m^−1^.^184^, ^185^ For the first time, GDY was employed as a hole transfer material to construct a photocathode for H_2_ production (**Figure** **17**a).^186^ Due to the strong π–π interactions between GDY and surface‐ligands (4‐mercaptopyridine) of QDs, CdSe QDs were loaded on GDY. Upon irradiation of 300 W Xe lamp (λ > 400 nm), the assembled photocathode exhibited nearly −70 µA cm^−2^ photocurrent in 0.1 m Na_2_SO_4_ (pH = 6.8) at an applied potential of 0 V versus NHE (Figure 17b). The Faradaic efficiency of H_2_ production up to 95% was obtained during a 12 h PEC measurement (Figure 17c). Photogenerated holes transferred to the counter electrode through the GDY layer were used for water oxidation, which could be confirmed by GC and Ocean Optics fluorescence based oxygen sensor. Therefore, this report demonstrated carbon materials could be used as a hole transporting layer to construct sensitized‐photocathode for water splitting, which provided us a novel idea to construct artificial photosynthetic systems.

**Figure 18:**
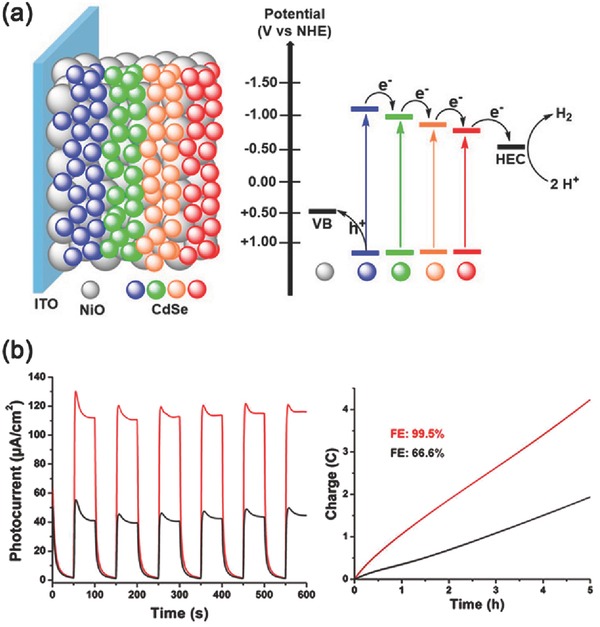
a) Schematic illustration of the rainbow photocathode and the process of interfacial electron transfer. b) Chopped‐light chronoamperometric measurements of the rainbow photocathodes with forward energetic gradient (red line) and reverse energetic gradient (black line) at applied bias potential (0 V vs Ag/AgCl) in air‐saturated buffer solution and the corresponding Faradaic efficiency of H_2_ evolution during a 5 h test. Reproduced with permission.^192^ Copyright 2017, American Chemical Society.

Very recently, Eisenberg and co‐workers had constructed a rainbow photocathode by spin‐coating different‐sized QDs in a sequentially layered manner, which demonstrated enhanced activity of PEC H_2_ production.^192^ As shown in **Figure** **18**a, the resulting rainbow photocathodes created an energetically favorable gradient for interfacial charge separation and subsequent electron transfer to a solution‐based hydrogen‐evolving catalyst, thus exhibiting good light‐harvesting ability and enhanced photoresponses compared with the reverse rainbow photocathodes under white LED light illumination. Under optimized conditions, a photocurrent density of as high as −115 µA cm^−2^ and a Faradaic efficiency of 99.5% for PEC H_2_ production were achieved (Figure 18b). This example demonstrates that rational arrangement of QDs with various sizes at electrode surface is an effective pathway to improve PEC efficiency.

## 4 Conclusion

PEC water splitting offers a great potential to provide sustainable and clean energy and has received great attentions in the past years. The above‐mentioned results demonstrate that sensitized‐photocathodes, either molecular dye‐ or quantum dot‐sensitized photocathodes, have showed extremely important application prospects for solar‐to‐fuel conversion via water splitting. With the aid of cocatalysts, the exploration of electrode construction and the modification of photosensitizers, both the efficiency and stability of PEC water‐splitting systems have been greatly enhanced. However, as an emerging research field with confused understanding of fundamental catalytic principles, and the lack of suitable electrode materials, sensitized photocathodes can only run in laboratory scale for PEC water splitting. We also have to admit that both molecular dyes and quantum dot working as photosensitizers have their own drawbacks. In terms of molecular dyes, poor light stability and instability on electrode surface are major disadvantages that limit their rapid development in dye‐sensitized photocathodes. Presently speaking, compared to molecular dyes counterparts, PEC systems based on QDs‐sensitized photocathodes have showed much better performance in H_2_ evolution, because of their inherent advantages in visible light response, exciton generation, charge separation, etc. However, the types of quantum dots used in sensitized photocathodes are very limited, and the loading amount of quantum dots on the electrode surface is low. Therefore, there is a long way to realize the practical application of sensitized photocathodes. And from our point of view, further efforts to improve the performance of such systems from the following aspects can be made.

i) Design and synthesis of light absorbers with exceptional solar‐light response. As the initial step, light absorption, especially the absorption of visible light, can greatly influence the photo‐electrocatalytic performance because light harvesting determines the theoretical uppermost efficiency for water splitting. Therefore, further exploitation of new and highly efficient visible‐light responsive materials should be made. As far as molecular dye photosensitizer is concerned, it is very important to design and develop molecular dyes with excellent visible‐light response. Moreover, in the design of molecular dyes, how to improve their stability and how to firmly anchor them onto the electrode surface are also of great significance. For the realm of colloidal quantum dots, it is very urgent to exploit new kinds of quantum dots with excellent visible‐light response, excellent photo stability, and low toxicity.
ii) Exploration of materials with excellent hole transfer properties. As a counterpart of photogenerated electrons, the capture and migration of photogenerated holes often determine the energy conversion efficiency of PEC system for its large barrier of migration. Up to date, less attention has been paid to this aspect. We think that in the future more and more attention should be paid to exploring new kinds of hole transfer materials for photocathode construction. At present, as the most frequently studied p‐type semiconductor material, NiO has a series of unique advantages as mentioned above. However, its poor hole transfer and unfavorable interfacial electron‐transfer kinetics affect the further improvement of PEC performance. Therefore, it is necessary to optimize the structure and composition of NiO semiconductor. In addition, although some other p‐type semiconductor materials, such as p‐Si and Cu_2_O, have also been developed, each of them has its own limitations such as photocorrosion or scarcity. So, developing new materials with excellent hole transfer capability, chemical stability and photostability, and also low cost are of great importance. In recent years, carbon materials have shown great potential applications in this field. Their well electrical conductivity, good carrier transport and separation capability, stability, and low cost have attracted wide attentions and researches.
iii) Development of highly efficient proton reduction cocatalysts and coupling with absorption units. In general, the introduction of the cocatalyst can significantly reduce the reaction active energy. So, developing more efficient and stable cocatalysts, especially non‐noble metal based cocatalysts, is also one of the key points of future research for photocathode construction. In addition, it is also one of the key points of future research to combine the cocatalyst and photosensitizers through reasonable design. The rational coupling between cocatalyst and photosensitizers can favor the capture of photoelectrons and the process of proton reduction. For dye sensitized photocathodes, the established coupling methods include covalent bond interaction, coordination bond interaction, ion connection, etc. The systems constructed by covalent bond interaction often show higher stability, while the building‐up process is usually complex. The other two methods, coordination bond interaction and ion connection, are easier to operate and more attentions should focus on how to further improve their stability and efficiency. As for quantum dots, because of their extremely small sizes, it is challenging to control preparation of photosensitizer‐catalyst coupling systems. Currently, few attentions have been paid to this aspect in the field of quantum dot sensitized photocathode. How to controllably synthesize QDs‐cocatalyst coupling systems and to efficiently anchor them onto electrode surface maybe one of the future research directions in sensitized PEC systems.
iv) Exploration of new methods for system construction. As a multi‐interface catalytic process, the catalytic efficiency of PEC water‐splitting system is affected by various interfaces. Therefore, it is very important to understand the kinetics of charges migration at the interface and to simplify the catalytic process of electrode interface, which can greatly promote the transfer and utilization of photogenerated electrons and holes. More importantly, a balance between the reduction and oxidation reactions should be achieved to avoid charge accumulation in the electrode, thus the stability of the PEC system can be improved.

The ultimate goal of building a mature photo‐electrochemical system is to achieve efficient bias‐free water splitting under solar‐light irradiation. Through this review, we can see that scientists have made unremitting efforts and achieved outstanding achievements in order to realize the above objectives. However, this field is still an on‐going research project and with both opportunities and challenges. Building more efficient and stable PEC systems are the goals pursued by researchers all the time.

## Conflict of Interest

The authors declare no conflict of interest.
